# Conflicts of Interest: Ayotte’s Response

**Published:** 2004-10

**Authors:** Pierre Ayotte

**Affiliations:** Public Health Research Unit, CHUQ-Laval University Medical Center, Quebec, Canada, E-mail: pierre.ayotte@inspq.qc.ca

Goozner contacted me by e-mail on 24 June 2004, and I promptly, honestly, and to the best of my knowledge answered questions regarding his claim that I did not disclose a conflict of interest while publishing our article (Bilrha et al. 2003) in *EHP*. Although I thought I made it clear that he was wrong in his allegations, he nevertheless chose to go ahead and include my name in a report published on the Center for Science in the Public Interest’s (CSPI) website (CSPI 2004) and in his letter to *EHP*. Here are the facts.

In our 2003 manuscript (Bilrha et al. 2003), we acknowledged funding from the Canadian Network of Toxicology Centers (CNTC). As the corresponding author, I read the conflict of interest statement and indicated that I had nothing to declare, neither for me nor for the coauthors. I did not know at that time that the Canadian Chemical Producer Association was partly funding the CNTC. On their website (CNTC 2004), the CNTC indicates being funded mostly by public sources (90%) and does not mention the identity of private sources. In any case, had I known this at the time of publication, it would not have changed anything, because I never personally received any funds (or compensation of any sort, or stood to gain financially) from the Canadian Chemical Producer Association or the Canadian Chlorine Coordinating Committee. Goozner is wrong when he mentions that several of my previous studies were funded by these interest groups. I was a coauthor on two articles published previously in *EHP*, in which funding from these interest groups was acknowledged, along with other sources of funding (Sandau et al. 2000, 2002). I collaborated in the work, but I was not the recipient of funds obtained from the Canadian Chemical Producer Association or the Canadian Chlorine Coordinating Committee. Funds from these organizations went to the principal investigator at Carleton University, and not a penny was transferred to me.

I truly believe that the authors of scientific manuscripts should disclose relevant conflicts of interest, and I support enforcing disclosure by appropriate means. However, because Goozner elected to choose the easy way by conducting an Internet-based research, without actually talking to me, he wrongly associated my name with scientific misconduct. I am presently seeking legal advice on this matter.
